# A Comparison of Dexmedetomidine-Propofol and Fentanyl-Propofol for Laryngeal Mask Airway Insertion: A Randomized Double-Blind Study

**DOI:** 10.7759/cureus.91713

**Published:** 2025-09-06

**Authors:** Nilima R Muthachen

**Affiliations:** 1 Department of Anaesthesiology, St Johns Medical College Hospital, Bangalore, IND

**Keywords:** dexmedetomidine, dexmedetomidine-propofol, fentanyl, fentanyl-propofol, laryngeal mask airway, muzis score, propofol

## Abstract

Introduction

Successful laryngeal mask airway (LMA) insertion requires suppression of airway reflexes and adequate jaw relaxation. Although propofol is the preferred induction agent, higher doses may cause adverse hemodynamic and respiratory effects. Adjuvants such as opioids or dexmedetomidine can enhance insertion conditions while minimizing propofol requirements. This pilot study compared dexmedetomidine-propofol and fentanyl-propofol combinations in terms of LMA insertion conditions and hemodynamic responses.

Materials and methods

In this prospective, randomized, double-blind pilot study, 40 American Society of Anesthesiologists (ASA) I and II patients were randomized to receive either an intravenous infusion of 1 microgram per kilogram of dexmedetomidine (Group D, n=20) diluted to 20 ml, or normal saline 20 ml (Group F, n=20) administered over 10 minutes. Following this, Group F received 1 microgram per kilogram of fentanyl over two minutes, while Group D received an equivalent volume of normal saline. All patients were induced with 1.5 mg/kg of propofol. The primary outcome was the ease of LMA insertion, assessed by jaw mobility and the presence of adverse airway reflexes (coughing, gagging, or movement during insertion), which was noted and scored. Secondary outcomes included heart rate (HR), systolic and mean arterial pressure, respiratory rate, and oxygen saturation, measured at baseline, pre-insertion, and at one, three, five, 10, 15, and 20 minutes post-insertion. Both the anesthesiologist administering the drugs and the investigator performing the insertion were blinded to group allocation.

Results

All patients in Group D had favorable LMA insertion scores (<2), while 30% in Group F had unfavorable scores (>2) (p=0.020). Apnea occurred in two patients in Group D and six patients in Group F. A statistically significant reduction in HR was observed in group D compared to group F; however, the values remained within the clinically acceptable range, without evidence of hemodynamic instability (HR before insertion (p<0.001), HR at one minute (p<0.001), HR at three minutes (p=0.026), HR at five minutes (p=0.022), and HR at 10 minutes (p=0.038)). Respiratory rate was significantly higher in Group D at the time points of one minute (p=0.003), three minutes (p=0.002), five minutes (p=0.011), 10 minutes (p=0.024), 15 minutes (p=0.007), and 20 minutes (p =0.002) post insertion.

Conclusion

When compared to fentanyl with propofol, dexmedetomidine with propofol provided effective conditions for LMA insertion with preserved respiratory function and comparable hemodynamic stability. Larger randomized studies are needed to confirm these findings and optimize dosing.

## Introduction

Insertion of a laryngeal mask airway (LMA) requires adequate mouth opening and suppression of upper airway reflexes such as coughing, gagging, and laryngospasm. Propofol is the preferred induction agent for LMA insertion because of its depressant effects on airway reflexes [1,2]. However, propofol is associated with several adverse effects, including hypotension, apnea, pain on injection, and excitatory movements [3]. Its respiratory depressant effect is dose-dependent and becomes more pronounced at higher doses [4].

To mitigate these limitations, adjuvant drugs such as opioids [5,6] or muscle relaxants [7,8] are often co-administered to reduce the required propofol dose and facilitate easier LMA insertion. However, opioids may predispose to respiratory depression, including apnea [9], while muscle relaxants can increase the risk of aspiration [10] and prolong recovery time [11].

Dexmedetomidine, a highly selective α2-adrenergic receptor agonist, possesses sedative, analgesic, and sympatholytic properties with minimal impact on respiratory function. Its intraoperative use has been shown to reduce anesthetic requirements, attenuate sympathetic responses to surgical stimulation, and facilitate faster recovery [12,13]. In addition, dexmedetomidine attenuates airway and circulatory responses during both intubation and extubation [14].

In this study, we compared the combination of propofol with dexmedetomidine to that with fentanyl, with particular focus on the ease of LMA insertion and associated hemodynamic changes.

## Materials and methods

This prospective, randomized, double-blind study was conducted after obtaining approval from the Institutional Ethics Committee and written informed consent from all participants. The study took place at Medical Trust Hospital, Kochi, India, between December 2011 and November 2012. A total of 40 patients were enrolled, all classified as ASA physical status I or II, aged 18 to 65 years, weighing between 30 and 100 kg, and having fasted for at least six hours for solids or two hours for clear fluids. The patients were scheduled for minor surgical procedures. A pre-anesthesia evaluation was performed on the day prior to surgery, during which relevant medical and surgical history, along with a detailed airway assessment, were recorded. Exclusion criteria included risk of aspiration, known allergy or sensitivity to propofol or volatile anesthetics, anticipated difficult airway (as assessed by Mallampati grade III/IV [15], restricted mouth opening, limited neck mobility, or short thyromental distance), significant medical comorbidities, or ASA physical status III or IV. Baseline assessments included age, gender, weight, and vital signs. 

Upon arrival in the operating room, standard monitors (ECG, pulse oximetry, and non-invasive blood pressure) were attached, and an intravenous line was established. Patients received supplemental oxygen via a facemask and were randomized into two groups based on computer-generated random numbers: Group D received an intravenous infusion of 1 microgram per kilogram of dexmedetomidine diluted to 20 ml over 10 minutes, while Group F received 20 ml of normal saline over the same duration, using a syringe infusion pump. Following this initial infusion, Group F received fentanyl at a dose of 1 microgram per kilogram diluted to 10 ml, over two minutes, while Group D received an equivalent volume of normal saline over the same duration. Thirty seconds later, anesthesia was induced with 1.5 mg/kg of propofol without any neuromuscular blocking agents. For the maintenance of anesthesia, 50% nitrous oxide and 1.5% sevoflurane in oxygen were used to provide a minimum alveolar concentration (MAC) of one. Ninety seconds after the completion of the propofol injection, the first attempt at LMA insertion was made, and jaw mobility was graded. During this 90-second period, patients received nitrous oxide in oxygen and a volatile anesthetic. The inhaled gases were given via face mask manually with assisted ventilation, allowing the patient to breath spontaneously. All insertions of LMA and grading of jaw mobility were done by the same blinded anesthesiologist. The LMA was selected according to the patient's weight.

The primary outcome was the ease of LMA insertion assessed using the modified Muzis score [16], and the secondary outcomes were hemodynamic changes including heart rate (HR), systolic blood pressure (SBP), mean arterial pressure, and respiratory rate (RR), compared between the dexmedetomidine-propofol and fentanyl-propofol groups. The modified Muzis score assessed jaw mobility (1: fully relaxed, 2: mild resistance, 3: tight but opens, 4: closed) and graded coughing or movement (1: none, 2: one or two coughs, 3: three or more coughs, 4: bucking or movement). Other events, such as spontaneous ventilation, breath-holding, and expiratory stridor, were also assessed. Scores below two in each category were deemed acceptable for LMA insertion. HR, SBP and mean blood pressure (MBP), RR, and oxygen saturation were monitored at baseline, just before insertion, and at one, three, five, 10, 15, and 20-minutes post-insertion.

If any movement occurred before or after LMA insertion, an additional dose of propofol (0.5 mg/kg) was administered. Bradycardia, defined as a HR of less than 40 beats per minute, was treated with 0.6 mg of atropine, while MBP below 60 mmHg was considered as hypotension and treated with a 6 mg bolus of ephedrine.

Patients were randomized into two groups using a computer-generated random number sequence. Allocation concealment was ensured with sequentially numbered, opaque, and sealed envelopes prepared by an independent investigator not involved in patient recruitment, drug preparation, or data collection. The anesthesiologist administering the study drugs, as well as the investigator performing LMA insertion and assessing jaw mobility, were blinded to group allocation. To maintain blinding, study drugs were prepared in identical syringes by a separate anesthesiologist who did not participate in patient management or outcome assessment.

As this was a pilot study, no formal sample size calculation was performed. A total of 40 patients (20 in each group) were included to generate local feasibility data, evaluate the methodology, and identify trends that could inform the design of larger, adequately powered randomized controlled trials (RCTs) in the future.

Demographic data were analyzed using the Chi-square test and Fisher's exact test. The Muzis scores and the incidence of apnea were assessed with Fisher's exact test, with P<0.05 considered significant. Hemodynamic variables were analyzed using Independent samples T test and repeated measure analysis of variance (ANOVA). All analyses were conducted using IBM SPSS Statistics for Windows, Version 13 (Released 2005; IBM Corp., Armonk, New York, United States).

## Results

A total of 40 patients were enrolled in the study. The two groups were comparable in terms of age, weight, and gender (Table 1).

**Table 1:** Demographic characteristics of the patient population (n=40) ^†^p-value calculated with Fisher's exact test; all other p values calculated using chi square test (p<0.05 significant); Data are mean ± SD (range) or number and percentage of patients; yrs: years; M: male; F: female; kg: kilogram; ASA: American Society of Anaesthesiologists; Group D: dexmedetomidine group, Group F: fentanyl group; n: number of patients; df: degree of freedom; CI: confidence interval.

	Group D (n=20)	Group F (n=20)	p-value	Statistic	df	Effect size (95% CI)
Age (yrs)	39.9 ± 14.6 (22-66)	44.0 ± 13.9 (18-62)	0.374	-0.9	38	-0.28 (-0.91, 0.34)
Gender (M/F) (%)	12/8 (60/40)	9/11 (45/55)	0.342	0.902	1	0.15 (-0.16, 0.43)
Weight (kg)	68.3 ± 11.2	64.2 ± 9.57	0.209	1.28	38	0.40(-0.23, 1.03)
Number of patients with apnea	2 (10%)	6 (30%)	0.235^†^	2.5	1	0.25 (-0.05,0.51)
ASA I/II (%)	12/8 (60/40)	11/9 (55/45)	0.749	0.102	1	0.05(-0.25, 0.35)

The Muzis score was favorable (less than two) for all patients in Group D. In Group F, the score was favorable in 14 patients and unfavorable (two or more) in six patients (30%), and this was statistically significant (p=0.020). In Group F, five patients had mild resistance to jaw mobility, and one patient had a tight jaw and coughing (Table 2).

Regarding episodes of apnea, six patients in Group F and two patients in Group D were affected; however, this difference was not statistically significant (p=0.235) (Table 2).

**Table 2:** Parameters for laryngeal mask airway insertion modified by Muzi and colleagues and other events *p<0.05 significant, all p values calculated using Fisher's exact test; Group D: dexmedetomidine group; Group F: fentanyl group; n= number of patients; df- degree of freedom; CI= confidence interval. Muzi et al. [16].

	Group D (n=20) (%)	Group F (n=20) (%)	p-value	Statistic	df	Effect size (95% CI)
Jaw mobility			0.02*	7.06	2	0.42 (0.18, 0.71)
1. Fully relaxed	20 (100%)	14 (70%)				
2. Mild resistance	0	5 (25%)				
3. Tight but opens	0	1 (5%)				
4. Closed	0	0				
Coughing, Movement			1	1.03	1	0.16 (0, 0.31)
1. None	20 (100%)	19 (95%)				
2. One or two coughs	0	1 (5%)				
3. Three or more coughs	0	0				
4. Bucking, movement	0	0				
Other events			0.235	2.5	1	0.25 (-0.05, 0.51)
Spontaneous ventilation	18 (90%)	14 (70%)				
Breath holding	0	0				
Expiratory stridor	0	0				
Tearing	0	0				
Muzis score - Median (Quartile 1, Quartile 3)	2 (2, 2)	2 (2, 3)	0.009*	140		0.03 (-0.05, 0.59)

LMA insertion was successfully achieved on the first attempt for all patients in Group D. In contrast, two patients in Group F required a second attempt and an additional bolus of propofol. However, there were no cases of failed LMA insertion in either group.

None of the patients in either group experienced expiratory stridor, breath-holding, or tearing. One patient in Group D had upper airway obstruction during infusion, which was resolved with a jaw thrust. Additionally, one patient in Group F developed hiccups during LMA insertion and required a total propofol bolus of 200 mg.

The baseline HR in Group D was 73.4±9.7 beats per minute, while in Group F, it was 84.7±13.5 beats per minute. Two patients in Group D experienced episodes of bradycardia (HR <40 beats per minute), which required treatment with atropine. In contrast, none of the patients in Group F exhibited bradycardia. Additionally, episodes of hypotension necessitating ephedrine boluses were observed in nine patients from Group F and four patients from Group D.

**Figure 1:**
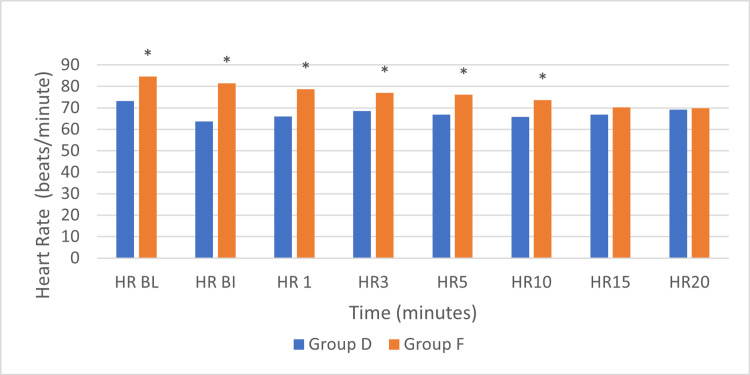
Heart rate (HR) in the two groups over time Group D: dexmedetomidine group; Group F: fentanyl group; HR BL: HR at baseline; HR BI: HR before insertion; HR1: HR at one minute after insertion; HR3: HR at three minutes after insertion; HR5: HR at five minutes after insertion; HR10: HR at ten minutes after insertion; HR15: HR at 15 minutes after insertion; HR20: HR at 20 minutes after insertion. *statistically significant difference between the two groups (p<0.05) (Independent samples T test); *HR BL (p=0.004), HR BI (p<0.001), HR 1 (p<0.001), HR 3 (p=0.026), HR 5 (p=0.022), HR 10 (p=0.038).

At baseline, HRs were significantly higher in Group F compared to Group D (p=0.004). Following the bolus, both groups showed a decrease in HR; however, the reduction was more pronounced in Group D. During the post-insertion period, Group D maintained consistently lower HR values, while Group F exhibited a gradual decline. Statistically significant differences persisted up to the 10-minute mark (HR before insertion (p<0.001), one minute after insertion (p<0.001), three minutes after insertion (p=0.026), five minutes after insertion (p=0.022), and 10 minutes after insertion (p=0.038)) but were no longer significant at 15 and 20 minutes (p=0.341 and 0.868, respectively) (Figure 1).

**Figure 2:**
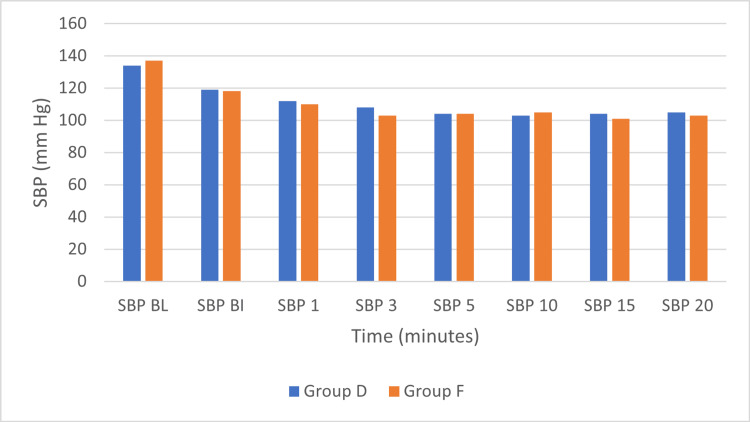
Systolic blood pressure (SBP) in the two groups over time Group D: dexmedetomidine group; Group F: fentanyl group; mmHg: millimeters of mercury; SBP BL: SBP at baseline; SBP BI: SBP before insertion; SBP 1: SBP at 1 minute after insertion; SBP 3: SBP at 3 minutes after insertion; SBP 5: SBP at 5 minutes after insertion; SBP 10: SBP at 10 minutes after insertion; SBP 15: SBP at 15 minutes after insertion; SBP 20: SBP at 20 minutes after insertion.

The baseline SBP and MBP at baseline were comparable between the two groups (SBP p=0.434; MBP p= 0.911). The mean SBP at baseline was comparable between Group D (134±11.3 mmHg) and Group F (137±14.1 mmHg), with no statistically significant difference (p=0.434). Following drug administration, SBP decreased in both groups over time. At each time point, before insertion (BI), and at one, three, five, 10, 15, and 20 minutes after insertion, the differences in SBP between the two groups remained statistically non-significant (all p>0.05). At five minutes, both groups had an identical mean SBP of 104 mmHg. These findings indicate that the changes in SBP over time were similar between the two groups, with no significant intergroup differences observed at any time point (Figure 2).

**Figure 3:**
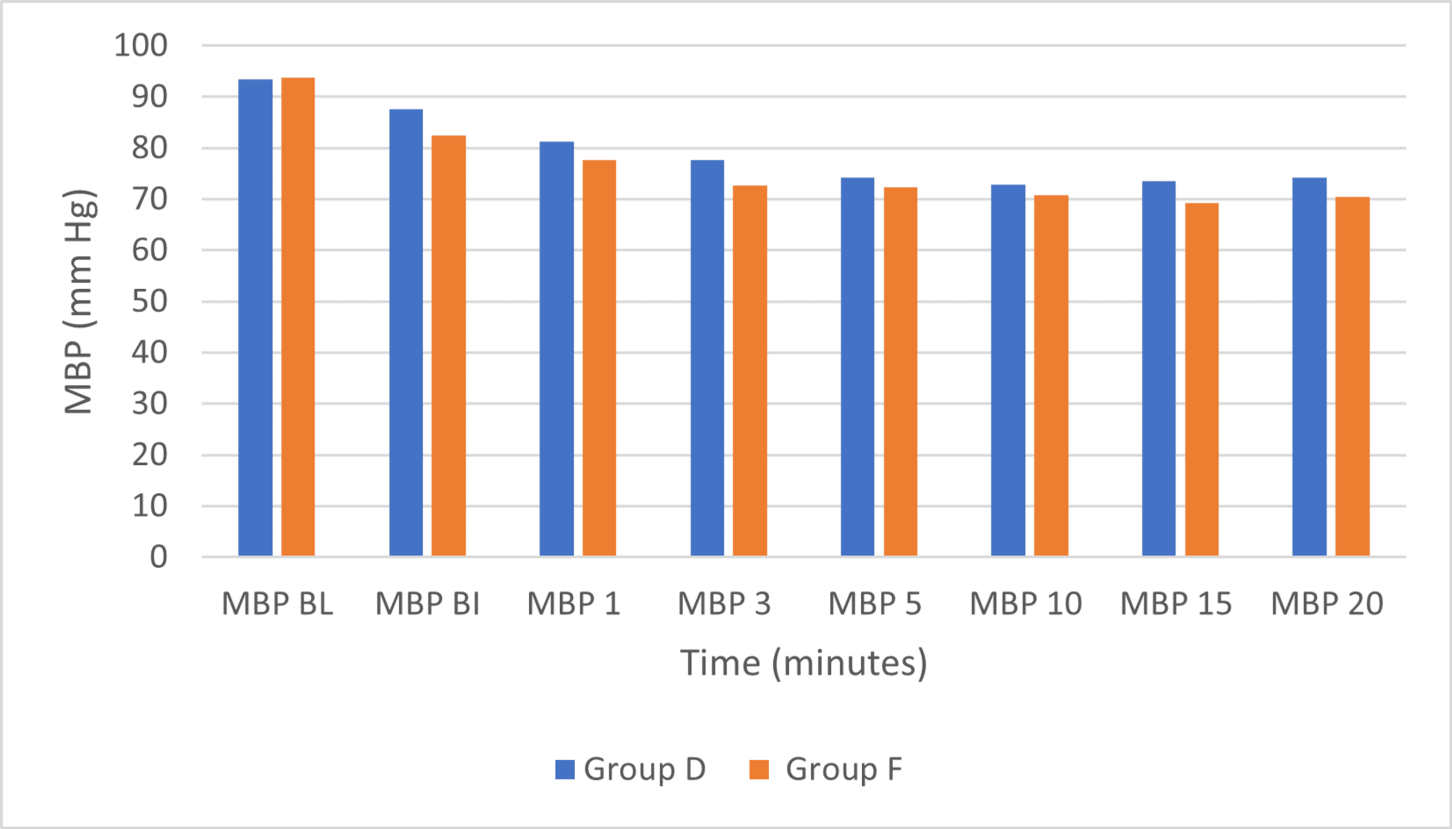
Mean blood pressure (MBP) in the two groups over time Group D: dexmedetomidine group; Group F: fentanyl group; mmHg: millimeters of mercury; MBP BL: MBP at baseline; MBP BI: MBP before insertion; MBP 1: MBP at 1 minute after insertion; MBP 3: MBP at 3 minutes after insertion; MBP 5: MBP at 5 minutes after insertion; MBP 10: MBP at 10 minutes after insertion; MBP 15: MBP at 15 minutes after insertion: MBP 20: MBP at 20 minutes after insertion.

In both Group D and Group F, there was a gradual decrease in MBP throughout the study period, which was not statistically significant (Figure 3).

The baseline mean respiratory rate (RR BL) was comparable between the two groups (Group D: 17.8±3.66 vs Group F: 16.7±4.09; p=0.376). Similarly, at the time before airway insertion (RR BI), no statistically significant difference was observed between them (Group D: 16.1±6.28 vs Group F: 12.7±7.3; p=0.117).

**Figure 4:**
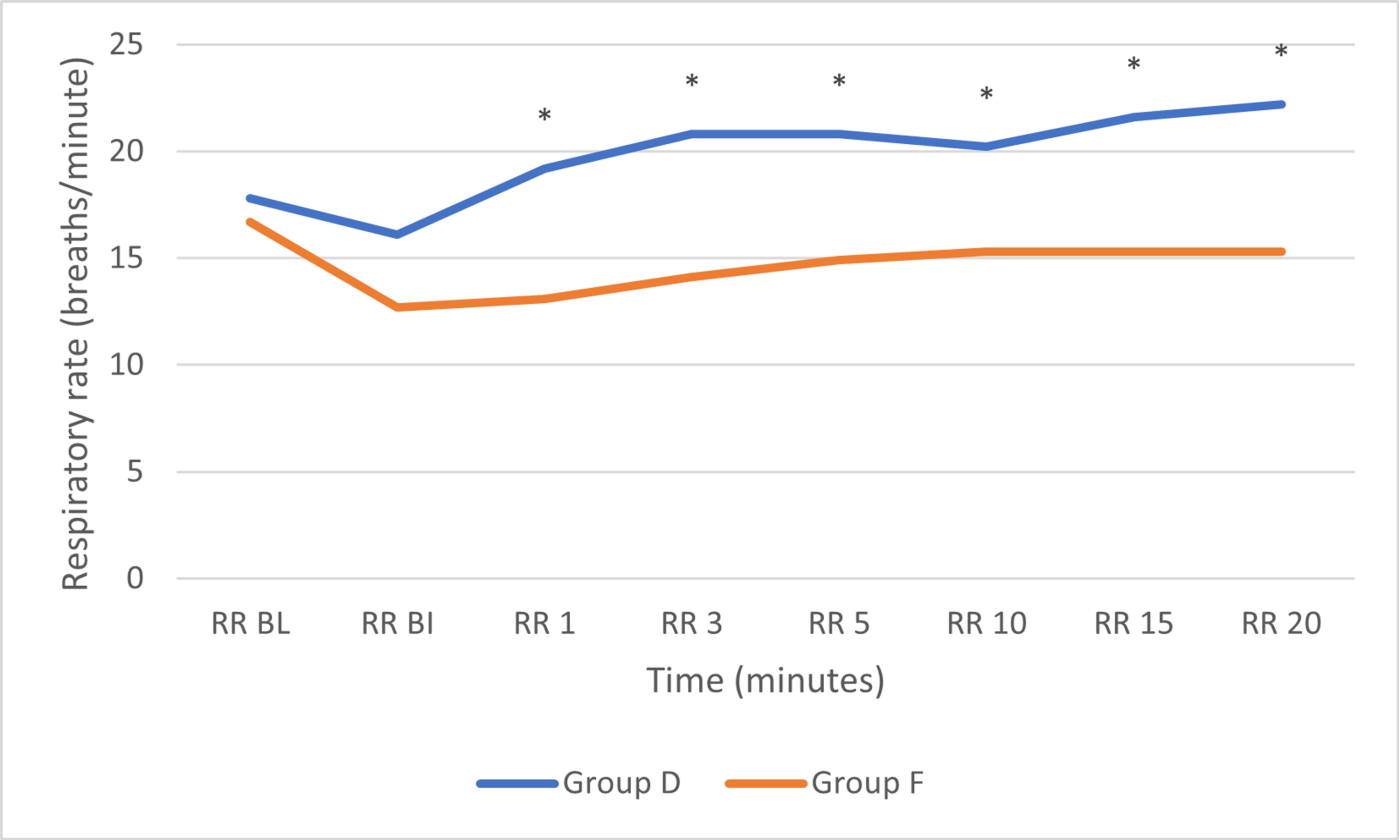
Respiratory rate (RR) in the two groups over time Group D: dexmedetomidine group; Group F: fentanyl group; min: minute; RR BL: RR at baseline; RR BI: RR before insertion; RR 1: RR at 1 minute after insertion; RR 3: RR at 3 minutes after insertion; RR 5: RR at 5 minutes after insertion; RR 10: RR at 10 minutes after insertion; RR 15: RR at 15 minutes after insertion; RR 20: RR at 20 minutes after insertion. *statistically significant difference between the two groups (p<0.05) (Independent samples T test); *statistically significant difference between groups (RR 1 (p=0.003); RR 3 (p=0.002); RR 5 (p=0.011); RR 10 (p=0.024); RR 15 (p=0.007); RR 20 (p=0.002))

However, starting from one minute after airway insertion, Group D consistently demonstrated a significantly higher RR compared to Group F at each recorded time point. Statistically significant differences were observed at one minute (p=0.003), three minutes (p=0.002), five minutes (p=0.011), 10 minutes (p=0.024), 15 minutes (p=0.007), and 20 minutes (p=0.002) after insertion (Figure 4).

## Discussion

The ease of LMA insertion depends on an anesthetic regimen that provides sufficient depth to suppress airway reflexes while maintaining hemodynamic stability. Propofol is the most commonly used agent for this purpose; however, when administered in higher doses as a sole anesthetic, it may cause cardiorespiratory depression and prolonged apnea. To overcome these limitations, various adjuvant drugs have been combined with propofol to facilitate LMA insertion.

Ghodki et al. demonstrated that dexmedetomidine could reduce the induction dose of propofol by up to 62.5%, thereby minimizing the hemodynamic perturbations associated with higher propofol doses [17]. Similarly, Zaballos et al. reported that co-administration of remifentanil reduced the propofol requirement for smooth insertion of LMA by approximately 60% [5]. These findings reinforce the role of adjuvant agents in reducing propofol requirements while maintaining favorable insertion conditions and hemodynamic stability.

Bergese et al. used dexmedetomidine as a bolus of 1 μg/kg over 10 minutes, followed by a continuous infusion of 0.7 μg/kg/h, and found that it provided satisfactory conditions for awake fibreoptic intubation [18]. Uzumcugil et al. compared 1 μg/kg of dexmedetomidine and 1 μg/kg of fentanyl with propofol for LMA insertion and reported comparable conditions, with dexmedetomidine preserving respiratory function better [19]. In contrast, our study demonstrated superior conditions for insertion with the dexmedetomidine-propofol combination, as all patients in Group D achieved a Muzi score less than two across categories, with no adverse airway events or need for second attempts. While insertion conditions in Group F were satisfactory and aligned with Uzumcugil et al., they were less favorable than those in Group D.

In our study, baseline and pre-insertion RRs were comparable between the two groups. However, patients in the dexmedetomidine group demonstrated significantly higher RRs compared with the fentanyl group at one minute, three minutes, five minutes, 10 minutes, 15 minutes, and 20 minutes after insertion. The incidence of apnea was also higher in Group F (six patients) compared with Group D (two patients), though this difference did not reach statistical significance, possibly due to the small sample size. Uzumcugil et al. reported a significant reduction in both the incidence and duration of apnea with dexmedetomidine [19]. Hsu et al. similarly observed that remifentanil, unlike dexmedetomidine, was associated with respiratory depression and apnea episodes [20]. These observations suggest that dexmedetomidine preserved spontaneous ventilation more effectively than fentanyl when used as a propofol adjuvant. While fentanyl is known to cause dose-dependent respiratory depression, dexmedetomidine exerts minimal effect on respiratory drive, which likely accounts for the observed difference. Taken together, these findings support the preferential use of dexmedetomidine in scenarios where preservation of spontaneous respiration is clinically advantageous.

A systematic review of seven RCTs, including 410 patients, by Ju et al. found that dexmedetomidine-assisted sedation improved the success rate of LMA insertion compared to controls. Additionally, RRs at five minutes were higher and the incidence of coughing was lower in the dexmedetomidine group [21]. These findings are consistent with our results, where dexmedetomidine provided better insertion conditions and better preservation of respiratory function compared to fentanyl.

Rustagi et al. [22] studied supraglottic airway insertion conditions with propofol induction after pre-treatment with dexmedetomidine or fentanyl and reported similar efficacy in facilitating insertion in both groups. In our study, there were no failed insertions in either group; all patients in the dexmedetomidine group (Group D) had successful insertions on the first attempt, whereas two patients in the fentanyl group (Group F) required a second attempt. Importantly, no adverse airway events were observed among the 40 patients studied. These findings suggest that both agents are effective adjuvants to propofol for supraglottic airway insertion, although significantly better jaw mobility was observed in the dexmedetomidine group (p=0.020).

Goertzen et al. [23] examined 28 anesthetic combinations used in 4,695 patients for general anesthesia induction and LMA insertion across 53 RCTs. They found that propofol combined with dexmedetomidine infused over 10 minutes ranked as the most effective regimen for reducing adverse outcomes, including apnea incidence and duration, airway adverse events, insertion failure, and inadequate anesthetic depth, during LMA insertion. Our study, which also employed a 10-minute dexmedetomidine infusion, supports these findings by demonstrating stable hemodynamics and favorable insertion conditions without adverse airway events.

Cakirgoz et al. [9] reported findings that were contrary to our study. In their study, 80 patients were randomized to receive either a 10-minute infusion of dexmedetomidine 1 μg/kg or remifentanil 2 μg/kg administered over 60 seconds before propofol induction for LMA insertion. They reported that remifentanil premedication was associated with more favorable hemodynamics, faster LMA insertion, and higher rates of optimal conditions compared with dexmedetomidine, although apnea duration was significantly shorter in the dexmedetomidine group [9]. These differences may be explained by variations in drug dosing, infusion protocols, or patient characteristics between the studies. In contrast, our study demonstrated that dexmedetomidine, when combined with propofol, provided stable hemodynamics and satisfactory insertion conditions without adverse airway events.

Studies have shown that both fentanyl and dexmedetomidine can cause bradycardia. Uzumcugil et al. [19] reported significant reductions in HR from baseline in both groups. In the fentanyl group, HR stabilized within one minute after LMA insertion, whereas in the dexmedetomidine group no significant change was detected between pre- and post-insertion values. Rustagi et al. [22] also reported that in their study, HR was significantly lower in the dexmedetomidine group. In our study, a significant decrease in HR persisted up to the 10 minute mark in Group D, suggesting that dexmedetomidine can cause sustained bradycardia. This may be of clinical importance in patients with low baseline HR.

Several studies indicate that both fentanyl and dexmedetomidine can affect blood pressure when used as anesthetic adjuvants. Ebert et al. [24] reported that dexmedetomidine reduced MBP, HR, and cardiac output in a dose-dependent manner. A meta-analysis by Ju et al. [21] showed that mean arterial pressure (MAP) in the dexmedetomidine group at one minute after catheterization was lower than in the control group, though the difference was not statistically significant. Zheng et al. [25] compared remifentanil (Group R), dexmedetomidine (Group D), and sufentanil (Group S) in patients undergoing radical gastrectomy and found that HR was significantly higher in Groups R and S than in Group D at multiple time points, with MAP also elevated in Group R. Dutt et al. [26] studied the efficacy and side effects of two doses of fentanyl for classical LMA insertion and found that higher doses produced a significant fall in systolic and mean arterial pressure, whereas lower doses provided a more stable hemodynamic profile. Çakırgöz et al. [9] reported that 2 μg/kg remifentanil before propofol induction led to greater suppression of MAP compared with a bolus of 1 μg/kg dexmedetomidine, although patients remained hemodynamically stable without increased risk of bradycardia or hypotension. Uzumcugil et al. [19] demonstrated comparable blood pressure effects between fentanyl and dexmedetomidine, both combined with propofol. In our study, no statistically significant changes in SBP or MBP were observed in either group. Although the overall decrease was minor, such changes may be clinically relevant in elderly patients or those with cerebrovascular disease. Sharma et al. [27] further showed that dexmedetomidine at a loading dose of 0.5 μg/kg was as effective as 1.0 μg/kg in reducing the propofol requirement, improving intubating conditions, and blunting the hemodynamic response to intubation, while being associated with fewer adverse effects such as hypotension and bradycardia.

In our study, a 10-minute infusion of dexmedetomidine 1 μg/kg was used for the initial bolus. Previous small studies, such as those by Uzumcugil et al. [19], have administered dexmedetomidine as a bolus. Both dexmedetomidine and fentanyl, when given 30 seconds before a propofol bolus, have been shown to provide adequate jaw relaxation and mouth opening 90 seconds after propofol injection. These waiting periods were derived from earlier work by Goyagi et al. [28], Uzumcugil et al. [19], and Tanaka et al. [29]. The fentanyl dose of 1 μg/kg was chosen based on these studies, balancing efficacy with safety and hemodynamic stability. Higher doses may further improve insertion conditions, but this requires confirmation in future dose-finding trials.

Several small studies have compared recovery patterns after the use of dexmedetomidine and opioids as adjuvants to propofol anesthesia. Uzumcugil et al. [19] reported significantly longer emergence times in the dexmedetomidine group. Similarly, a systematic review and meta-analysis by Song et al. [30], which included 11 RCTs with 801 patients, found that dexmedetomidine use was associated with delayed extubation or LMA removal. In our study, dexmedetomidine was administered only up to LMA insertion; therefore, recovery profiles were not analyzed. Nonetheless, emergence time may be a clinically relevant consideration, particularly for short-duration or outpatient procedures.

The main limitation of this study is the small sample size, as it was designed as a pilot to generate local data and assess feasibility. The limited number of participants may have reduced the statistical power to detect subtle differences, particularly in the hemodynamic variables, which appeared comparable between groups. Larger, adequately powered studies are therefore needed to confirm these preliminary observations. Another limitation is the absence of a control group receiving propofol alone. This was intentional, as prior studies have demonstrated that propofol doses sufficient for LMA insertion may compromise hemodynamic stability and respiration. Baseline HRs were also not statistically comparable between groups, which may have introduced bias. A larger sample size could help minimize the impact of such imbalances. Finally, sevoflurane was administered prior to LMA insertion in both groups to standardize conditions. Its use may have influenced LMA tolerance, potentially underestimating the drug requirements in both groups. In addition, as this study was not designed to evaluate optimal dosing, it remains possible that higher doses of fentanyl could produce insertion conditions similar to those achieved with dexmedetomidine.

## Conclusions

In this pilot randomized trial, dexmedetomidine combined with propofol provided favorable conditions for LMA insertion and appeared to be a suitable alternative to fentanyl as a propofol adjuvant. Both regimens maintained comparable hemodynamic stability, while dexmedetomidine was associated with a better preservation of respiratory function. These preliminary observations require confirmation in larger, adequately powered randomized trials to refine dosing strategies and improve generalizability. A dose-finding study to establish the optimal fentanyl dose with propofol in our population is warranted. In addition, larger trials evaluating emergence characteristics are recommended to support wider use in outpatient surgeries.
